# IL-8 is a potential biomarker for retinal detachment secondary to choroidal melanoma

**DOI:** 10.7717/peerj.21183

**Published:** 2026-05-28

**Authors:** Zhen Xing, Xinran Zhang, Wei Yu, Yingqing Lei, Guiqu Wang, Hongbin Lv

**Affiliations:** Department of Ophthalmology, The Affiliated Hospital of Southwest Medical University, Sichuan, China

**Keywords:** Choroidal melanoma, Retinal detachment, Interleukin-8, Inflammatory factors, Mendelian randomisation

## Abstract

This study aims to identify potential biomarkers for retinal detachment secondary to choroidal melanoma (CM). Mendelian randomisation (MR) analysis, immunohistochemistry, cell proliferation and migration assays, co-culture experiments involving human microglial cells (HMC3) and CM cells, and tube formation assay were employed to validate the role of IL-8 in retinal detachment secondary to choroidal melanoma. Mendelian randomisation analysis identified four inflammatory mediators causally linked to malignant melanoma progression: CDCP1 concentration (*P* = 0.017, OR = 0.999, 95% CI [0.998–1.000]), CSF-1 concentration (*P* = 0.020, OR = 1.002, 95% CI [1.000–1.003]), IL-10Rβ concentration (*P* = 0.004, OR = 0.999, 95% CI [0.998–1.000]), IL-17C concentration (*P* = 0.002, OR = 1.003, 95% CI [1.001–1.005]); Four inflammatory factors exhibited a causal relationship with retinal detachment progression: IL-15Rα concentration (*P* = 0.029, OR = 1.001, 95% CI [1.000–1.001]), IL-2Rβ concentration (*P* = 0.007, OR = 1.003, 95% CI [1.001–1.004]), IL-8 concentration (*P* = 0.045, OR = 1.002, 95% CI [1.000–1.004]), TSLP concentration (*P* = 0.028, OR = 1.002, 95% CI [1.000–1.004]). MR analysis indicated that genetically predicted IL-8 levels are associated with an increased risk of retinal detachment. IL-8 is highly expressed in CM tissues and promotes the proliferation of CM and HMC3 cells. It partially relieves the inhibitory effects mediated by the supernatant of HMC3 cells and promotes angiogenesis. IL-8 may be involved in CM-related inflammatory microenvironments and secondary exudative retinal detachment. It is a potential biomarker and provides a new target for targeted anti-inflammatory therapy and improved patient prognosis.

## Introduction

**Figure 1:**
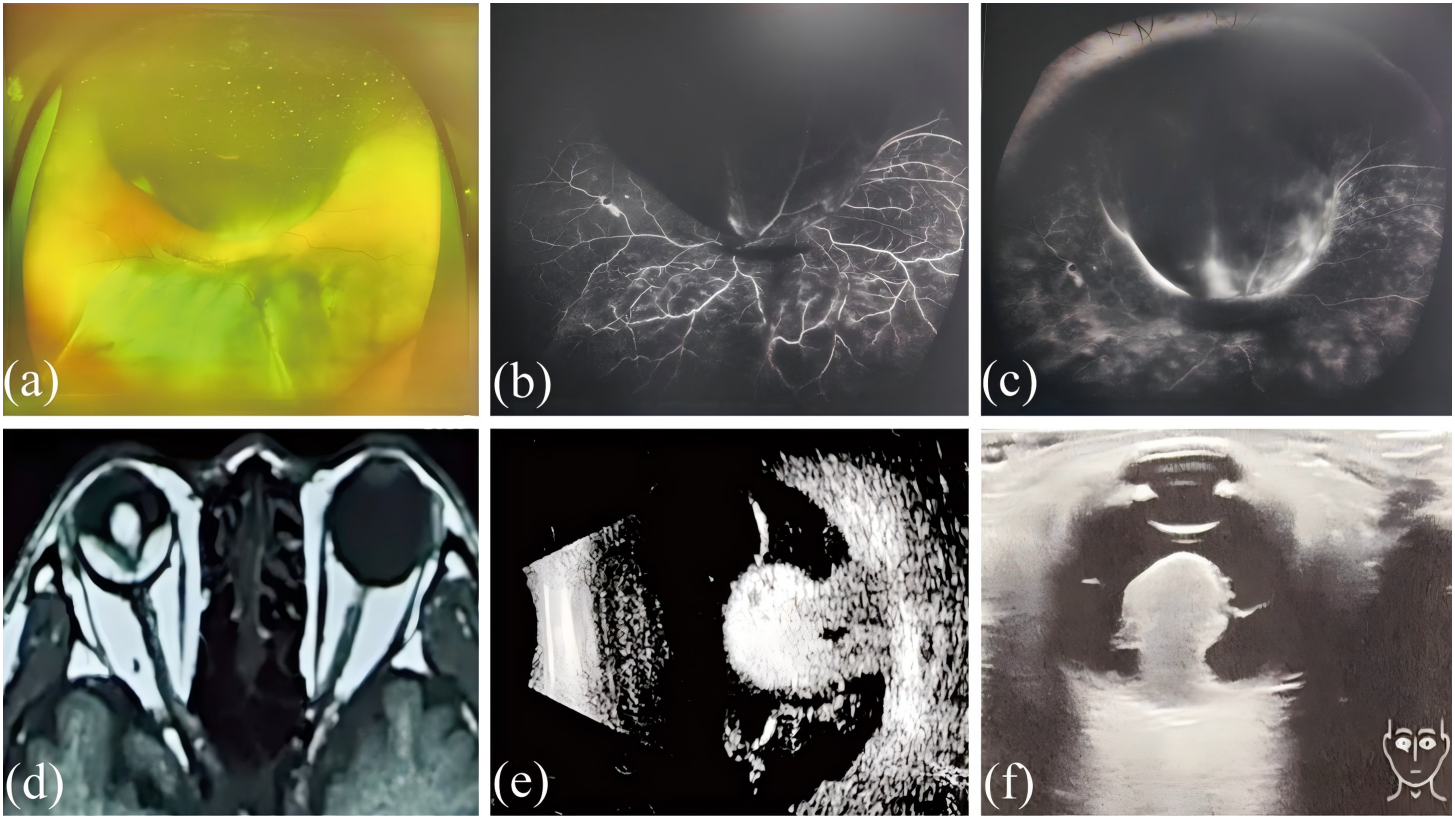
Imaging pictures of a patient with choroidal melanoma secondary to retinal detachment.

Malignant melanoma is a highly aggressive tumor with a gradually increasing global incidence, most commonly seen in Caucasian Europeans, often occurring in areas such as the skin and mucous membranes (Maida et al., 2019), but can also be seen in the eye, for example, choroidal melanoma (CM) is the most common intraocular primary malignancy in adults (Gelmi & Jager, 2024). Retinal detachment is one of the common blinding eye diseases, and exudative retinal detachment is often due to inflammation, vascular abnormalities, or tumors, which cause exudate that exceeds the osmotic pump function of the RPE cells to accumulate in the subretina, causing a separation between the neural epithelial and pigment epithelial layers of the retina (Lin et al., 2024). Choroidal melanoma can cause pathological processes such as increased vascular osmotic pressure, accumulation of inflammatory factors, and exudation of tissue fluid, which can be secondary to exudative retinal detachment, ultimately leading to severe vision loss and even life-threatening (Xie et al., 2023; Zhao et al., 2023). Figure 1 shows the ocular imaging images of patients with choroidal melanoma secondary to retinal detachment collected in clinical work, and we found that many patients with choroidal melanoma secondary to retinal detachment have a very poor visual prognosis, and there is currently no better treatment (Godtfredsen, 1949; Anguita et al., 2025).

Inflammatory factors may be important players in the disease progression of choroidal melanoma secondary to retinal detachment, but there is a lack of genetic evidence for their causal associations. Lu et al. (2024) found that in a two-sample Mendelian randomization study of 41 inflammatory factors (not exclusively included in the 91 inflammatory factors in the present study) and malignant melanoma, IL-16, IL-8, MCP-1 and TGF-β. These four inflammatory factors are risk factors for malignant melanoma, but further additions and explorations are needed due to the small number of inflammatory factors. Fortes et al. (2023) found that high levels of NLR and d-NLR were independently associated with an increased risk of 10-year melanoma mortality. Fruntelată et al. (2023) found that in malignant melanoma, chronic inflammation is mediated by the production of inflammatory molecules (cytokines, chemokines, prostaglandins), which are formed to favor carcinogenesis, tumor invasion and metastasis. In addition, Shah et al. (2020) found that inflammation is a key predictor for the development of exudative retinal detachment. Liu et al. (2023) found that VEGF, MCP-1, IL-8 and IL-6 levels were elevated in atrial fluid of patients with exudative retinal detachment. Ferreira et al.’s (2023) research indicates that inflammatory mediators circulating throughout the body can induce ocular inflammatory responses *via* the blood-ocular barrier. Given the choroid’s rich blood supply, inflammatory mediators in the bloodstream can reach the choroid and provide a favourable microenvironment for tumour development. Identifying key inflammatory factors that influence the progression of malignant melanoma and retinal detachment from a genetic perspective, and validating them through experiments, could offer more targeted therapeutic guidance for patients.

Mendelian randomization, leveraging genetic variants as instrumental variables, offers greater scientific rigor than traditional observational studies. This study aims to conduct the first two-sample Mendelian randomization analysis of 91 circulating inflammatory factors and their causal relationships with malignant melanoma and retinal detachment. It will also explore how these factors relate to retinal detachment caused by choroidal melanoma. Furthermore, experiments will verify the role of IL-8, a common pathogenic factor for malignant melanoma and retinal detachment, in choroidal melanoma. The findings will provide a theoretical basis for targeted therapy.

## Materials and Methods

### Mendelian randomization

#### Study design

This study was based on the analysis of genome-wide association study (GWAS) published data on malignant melanoma, retinal detachment, and inflammatory factors. Inflammatory factors were analyzed as the exposure factors, significantly associated single nucleotide polymorphisms (SNPs) were selected as the instrumental variables (IVs), and malignant melanoma and retinal detachment were used as the two endpoint variables, respectively. To improve the reliability of the results of MR analysis, the following three assumptions should be met: firstly, IVs are strongly associated with circulating levels of inflammatory factors; secondly, no confounding factors are associated with malignant melanoma and retinal detachment; and lastly, IVs affect the outcome only through exposure, and there are no other causal pathways to influence the outcome.

#### Genome-wide association summary data

The summary statistics for this study were obtained from the GWAS summary dataset, where exposure factors were derived from the GWAS catalog database (https://www.ebi.ac.uk/gwas/publications/37563310) of genetic data for 91 inflammatory factors recruiting 14,821 European subjects, no. GCST90274758–GCST90274848, and genetic data for two outcome factors, malignant melanoma and retinal detachment, from the GWAS database (https://gwas.mrcieu.ac.uk/datasets/ukb-b-12915 and https://gwas.mrcieu.ac.uk/datasets/ukb-b-8637) with numbers UKB-b-12915 and UKB-b-8637, respectively. As this study was based on publicly available data, no additional ethical approval was required. The 91 inflammatory factors were normalized by inverse transformation. Considering the limited sample size and number of SNPs, the association threshold was adjusted to *P* < 1.0 × 10^−6^ when selecting SNPs associated with inflammatory factors. In addition, the effect of linkage disequilibrium among SNPs was mitigated by using linkage disequilibrium for the selected SNPs (Clump parameter: r^2^ < 0.001, Genetic Distance <10,000 kb) to ensure that each exposure IVs independence between them.

#### Selection of instrumental variables

MR analysis was performed in this study using R.2024.12.1.0 and the “TwoSample MR” software package. The F statistic was calculated, and F > 10 was considered as a strong instrumental variable, indicating the absence of weak IVs bias, and the intermediate allele frequencies of the palindromic SNPs were excluded, and the SNPs that met the criteria were screened out.

#### Statistical analyses

In this study, MR analysis was performed using conventional analysis methods, including five methods, including MR-Egger regression, weighted median estimation model (WME), inverse variance weighted (IVW), weighted model, and simple model, *etc*. The IVW method is an aggregated SNPs Wald ratio estimation, the main statistical method for estimating causal effects by weighted average of effect estimates of IVs, which demonstrated the highest rank of statistical power. Therefore, it was used as the primary method for assessing causality in this study. In addition, the other four were used as secondary analysis methods, and when the direction of the results of these four models was consistent with the main analysis method, they were considered to be causal associations for stabilization.

Sensitivity analyses were performed using the leave-one-out method, the Cochran Q-test and the MR-Egger regression analysis to verify the robustness of the results. The leave-one-out method was utilized in order to determine and deep MR results whether they were affected by individual SNPs, to observe whether the effect value in the cause of a large change and the sensitivity value. In addition, IVW and MR-Egger’s Q statistic were utilized to estimate the heterogeneity between SNPs. *P* > 0.05 in Cochran’s Q test indicated that there was no heterogeneity between IVs. Finally, the presence of horizontal pleiotropy was assessed using MR-Egger regression with an intercept correlation of *P* > 0.05 indicating the absence of horizontal pleiotropy.

### Experimental validation

Six patients with choroid-derived retinal detachment and six patients undergoing enucleation due to non-choroidal conditions such as ocular trauma were enrolled. Immunohistochemical analysis was performed on tumour histopathology sections and choroidal tissue sections from each group to compare IL-8 expression levels. Cell experiments were conducted using CM tumor cells (MUM-2B and M619), human microglial cells (HMC3), and human retinal microvascular endothelial cells (HRMECs). All four cell lines were obtained from BNCC Biotechnology Co., Ltd. in China.

#### Immunohistochemistry

Non-tumor/tumor choroidal tissues fixed in 4% paraformaldehyde were dehydrated, embedded in paraffin at 56 °C for 2 h, sectioned at 4 μm, and mounted on slides. After dewaxing and drying, endogenous peroxidase was blocked, and tissues were incubated with IL-8 (1:100) primary antibody overnight at 4 °C, followed by secondary antibody for 1 h. DAB staining, hematoxylin counterstaining and mounting were performed, and images were analyzed using ImageJ.

#### CCK-8 assay

The growth rates of cells were compared between the IL-8 intervention group (with IL-8 concentrations of 100, 400, and 800 ng/ml) and the non-IL-8 intervention group. MUM-2B, M619 and HMC3 cells were respectively seeded in a 96-well plate. After overnight incubation, 10 μL of CCK8 solution was added to each well and incubated for 1 h. The absorbance at 450 nm was detected by a microplate reader, with the absorbance value representing cell viability. Each group of cells was repeated in three wells for each experiment, and the experiment was independently repeated three times.

#### Transwell assays

The migratory capacity of cells was compared between the non-IL-8 intervention group and the IL-8 intervention group (800 ng/ml). Cell suspensions of MUM-2B, M619 and HMC3 cells were prepared at a concentration of 5 × 10^4^ cells/mL using serum-free culture medium. A volume of 200 μL of the cell suspension was added to the upper chamber of a Transwell insert, and 800 μL of culture medium containing 10% fetal bovine serum was added to the lower chamber. The cells were cultured at room temperature for 24 h. The insert was then removed, and the cells that had not migrated through the pores were removed from the upper surface of the membrane. The membrane was fixed with paraformaldehyde for 15 min and stained with 0.1% crystal violet for 15 min. After washing with PBS, the number of migrated cells was observed and counted under an inverted microscope to reflect the migratory capacity of the cells. Each group of cells was repeated in three wells, and the experiment was independently repeated three times.

#### Cell co-culture experiment

After culturing CM cells and HMC3 cells separately in appropriate growth media for 24 h, collect the cell supernatants. Seed CM cells into a 96-well plate. Three groups were established: Group A contained MUM-2B and M619 cells supplemented with the original culture medium; Group B contained MUM-2B and M619 cells supplemented with HMC3 cell supernatant; Group C contained MUM-2B and M619 cells supplemented with HMC3 cell supernatant and concurrently treated with IL-8 (800 ng/ml). After 24 h of culture, CCK-8 assays were performed to assess growth rate changes across all groups. Each group of cells was repeated in three wells for each experiment, and the experiment was independently repeated three times.

#### Tube formation assay

Matrigel matrix was added to a prechilled 24-well plate. After the matrix was evenly spread, the plate was incubated at 37 °C for 2 h. A cell suspension of HRMECs was prepared and added to the wells containing the matrix at a volume of 300 μL per well, with 50,000 cells per well. The experiment was divided into four groups. Two hundred microliters of PBS, CM cell supernatant, CM cell supernatant with 50 ng/ml IL-8, or CM cell supernatant with 100 ng/ml IL-8 was added to the cell suspension in each group, respectively. The angiogenesis was compared after 12 h. Each group of cells was repeated in three wells, and the experiment was independently repeated three times.

#### Data analysis

Experimental results were analysed and statistically evaluated using ImageJ and GraphPad Prism 10.0 software. Statistical significance of intergroup differences was assessed *via* one-way analysis of variance (ANOVA) and independent samples t-tests, with *P* < 0.05 indicating statistically significant differences.

### Ethics

Approved by the Ethics Committee of Clinical Trials of the Affiliated Hospital of Southwest Medical University (approval No.: KY2025169), this study meets the relevant ethical requirements. This study has obtained informed consent from the participants, and written informed consent forms have been acquired.

## Results

### Causal association of inflammatory factors with malignant melanoma

**Figure 2:**
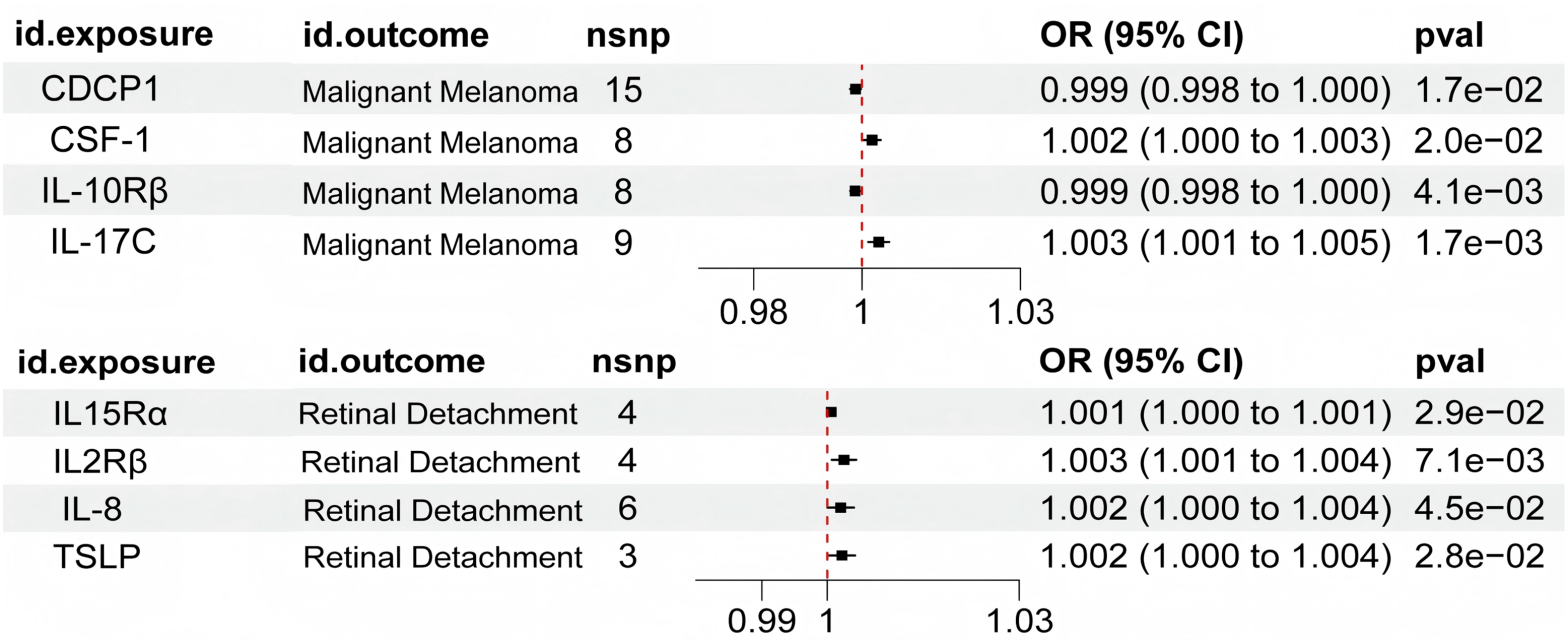
Forest plot for MR analysis of inflammatory factors (IVW method).

**Figure 3:**
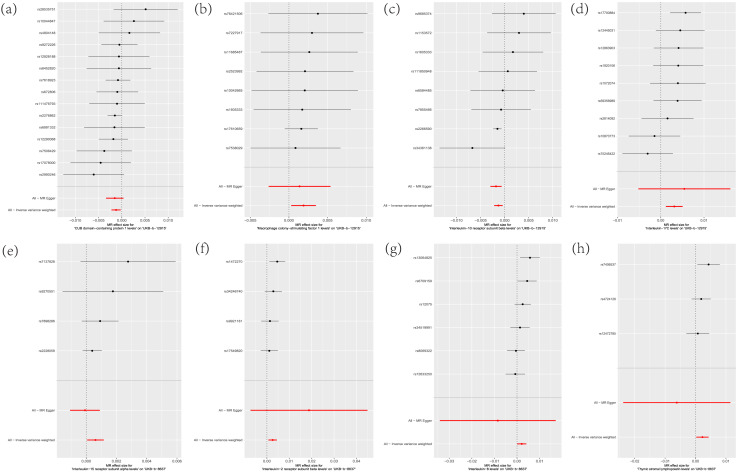
Scatterplot of MR analysis. (A) CDCP1; (B) CSF-1; (C) IL-10Rβ; (D) IL-17C; (E) IL-15Rα; (F) IL-2Rβ; (G) IL-8; (H) TSLP.

**Figure 4:**
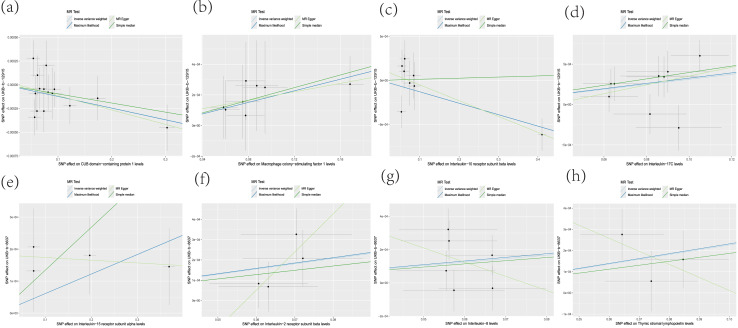
Forest map for MR analysis. (A) CDCP1; (B) CSF-1; (C) IL-10Rβ; (D) IL-17C; (E) IL-15Rα; (F) IL-2Rβ; (G) IL-8; (H) TSLP.

The minimum F-value of SNP was 20.84, indicating that the IVs had suitable robustness and strength of association. Forest plot after applying Bonferroni correction showed that there were four inflammatory factors CUB structural domain containing protein 1(CDCP1) concentration (*P* = 0.017, OR = 0.999, 95% CI [0.998–1.000]), macrophage colony-stimulating factor-1 (CSF-1) concentration (*P* = 0.020, OR = 1.002, 95% CI [1.000–1.003]), interleukin 10 receptor beta (IL-10Rβ) concentration (*P* = 0.004, OR = 0.999, 95% CI [0.998–1.000]), interleukin-17C (IL-17C) concentration (*P* = 0.002, OR = 1.003, 95% CI [1.001–1.005]) showed an association with malignant melanoma (*P* < 0.05), as shown in Fig. 2. The results suggest that both CSF-1 and IL-17C were able to increase the risk of malignant melanoma, and CDCP1 and IL-10Rβ were able to decrease the risk of malignant melanom (Table 1, Figs. 3 and 4).

**Table 1:** Five MR analyses of inflammatory factors and malignant melanoma and retinal detachment.

Exposure factors	Outcome factors	Methods	*P* value	95% CI	OR
CDCP1	Malignant melanoma	IVW	0.017	[0.998–1.000]	0.999
		MR-Egger	0.140	[0.997–1.000]	0.998
		WME	0.065	[0.997–1.000]	0.999
		Simple mode	0.397	[0.997–1.001]	0.999
		Weighted mode	0.053	[0.997–1.000]	0.999
CSF-1	Malignant melanoma	IVW	0.041	[1.000–1.003]	1.002
		MR-Egger	0.347	[0.998–1.006]	1.002
		WME	0.082	[1.000–1.004]	1.002
		Simple mode	0.168	[0.999–1.005]	1.002
		Weighted mode	0.147	[1.000–1.004]	1.002
IL-10Rβ	Malignant melanoma	IVW	0.002	[0.998–0.999]	0.999
		MR-Egger	0.031	[0.997–0.999]	0.998
		WME	0.001	[0.998–0.999]	0.999
		Simple mode	0.581	[0.996–1.002]	0.999
		Weighted mode	0.014	[0.998–0.999]	0.999
IL-17C	Malignant melanoma	IVW	0.002	[1.001–1.005]	1.003
		MR-Egger	0.348	[0.995–1.016]	1.005
		WME	0.002	[1.001–1.007]	1.004
		Simple mode	0.069	[1.000–1.008]	1.004
		Weighted mode	0.034	[1.000–1.007]	1.003
IL-15Rα	Retinal detachment	IVW	0.029	[1.000–1.001]	1.001
		MR-Egger	0.882	[0.999–1.001]	1.000
		WME	0.057	[1.000–1.001]	1.001
		Simple mode	0.182	[1.000–1.002]	1.001
		Weighted mode	0.197	[1.000–1.001]	1.001
IL-2Rβ	Retinal detachment	IVW	0.007	[1.001–1.004]	1.003
		MR-Egger	0.294	[0.993–1.046]	1.019
		WME	0.092	[1.000–1.004]	1.002
		Simple mode	0.473	[0.998–1.005]	1.001
		Weighted mode	0.441	[0.998–1.005]	1.001
IL-8	Retinal detachment	IVW	0.045	[1.000–1.004]	1.002
		MR-Egger	0.550	[0.996–1.017]	0.992
		WME	0.147	[0.999–1.004]	1.002
		Simple mode	0.721	[0.997–1.004]	1.001
		Weighted mode	0.645	[0.997–1.004]	1.001
TSLP	Retinal detachment	IVW	0.028	[1.000–1.004]	1.002
		MR-Egger	0.618	[0.977–1.012]	0.994
		WME	0.172	[0.999–1.004]	1.002
		Simple mode	0.487	[0.998–1.005]	1.001
		Weighted mode	0.422	[0.999–1.004]	1.001

**Figure 5:**
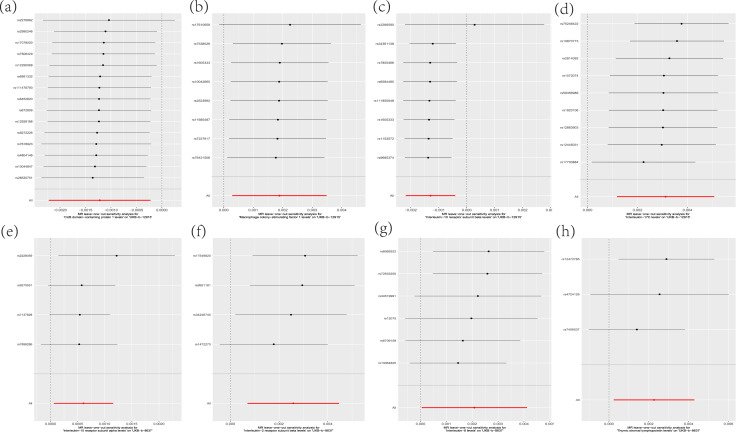
Sensitivity analysis of the leave-one-out method. (A) CDCP1; (B) CSF-1; (C) IL-10Rβ; (D) IL-17C; (E) IL-15Rα; (F) IL-2Rβ; (G) IL-8; (H) TSLP.

Leave-one-out analysis was performed by analyzing CDCP1, CSF-1, IL-10Rβ and IL-17C related SNPs, and it was found that the effect and total effect sizes of the included IVs were relatively close to each other, and SNPs with a large impact on the causal association estimates had not been identified (Fig. 5).

**Figure 6:**
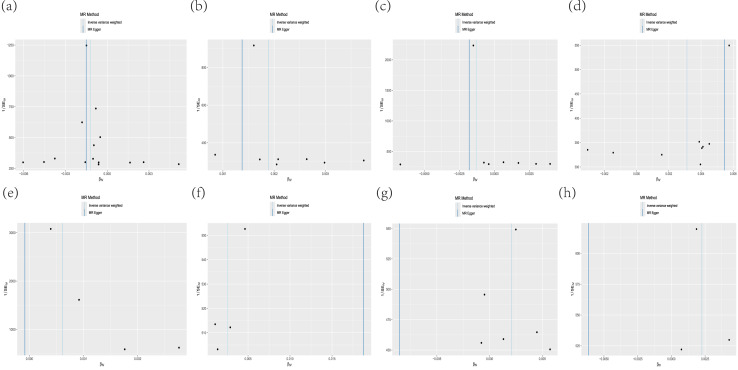
Heterogeneity test funnel plot. (A) CDCP1; (B) CSF-1; (C) IL-10Rβ; (D) IL-17C; (E) IL-15Rα; (F) IL-2Rβ; (G) IL-8; (H) TSLP.

The MR-Egger method yielded *P*-values of 0.73, 1.00, 0.37 and 0.24, while the IVW method produced *P*-values of 0.79, 1.00, 0.33 and 0.31. For CDCP1, CSF-1, IL-10Rβ and IL-17C (*P* > 0.05) indicated no heterogeneity, as shown in Table 2. This was further confirmed by the funnel plot (Fig. 6). Furthermore, the MR-Egger regression analysis yielded *P*-values of 0.73, 0.79, 0.27 and 0.67 respectively, all exceeding 0.05. This supports the absence of pleiotropy among SNPs and indicates that the instrumental variables do not exert effects through other confounding exposures (Table 2).

**Table 2:** Quality control results of the causal relationship between inflammatory factors and malignant melanoma and retinal detachment, respectively.

Exposure factors	Outcome factors	Heterogeneity			Pleiotropy	
		Methods	Q	*P* value	Eagger-intercept	*P* value
CDCP1	Malignant melanoma	IVW	9.671	0.786	3.67E−05	0.729
		MR-Egger	9.545	0.731		
CSF-1	Malignant melanoma	IVW	0.681	0.998	5.53E−05	0.793
		MR-Egger	0.606	0.996		
IL-10Rβ	Malignant melanoma	IVW	8.050	0.328	0.0001	0.269
		MR-Egger	6.457	0.374		
IL−17C	Malignant melanoma	IVW	9.442	0.306	−0.0002	0.674
		MR-Egger	9.190	0.239		
IL-15Rα	Retinal detachment	IVW	2.952	0.399	0.0002	0.239
		MR-Egger	0.203	0.904		
IL-2Rβ	Retinal detachment	IVW	2.274	0.518	−0.0011	0.347
		MR-Egger	0.787	0.675		
IL-8	Retinal detachment	IVW	7.456	0.189	0.001	0.462
		MR-Egger	6.398	0.171		
TSLP	Retinal detachment	IVW	1.838	0.399	0.001	0.519
		MR-Egger	0.949	0.330		

### Causal association between inflammatory factors and retinal detachment

The minimum F-value of SNP was 20.84, indicating suitable robustness and strength of association for IVs. Forest plot after applying Bonferroni correction showed four inflammatory factors interleukin-15 receptor alpha (IL-15Rα) concentration (*P* = 0.029, OR = 1.001, 95% CI [1.000–1.001]), interleukin 2 receptor beta (IL-2Rβ) concentration (*P* = 0.007, OR = 1.003, 95% CI [1.001–1.004]), interleukin 8(IL-8) concentration (*P* = 0.045, OR = 1.002, 95% CI [1.000–1.004]), and thymic stromal lymphopoietin (TSLP) concentration (*P* = 0.028, OR = 1.002, 95% CI [1.000–1.004]) showed an association with malignant melanoma (*P* < 0.05) (Fig. 2). The results suggest that IL-15Rα, IL-2Rβ, IL-8 and TSLP are all capable of increasing the risk of developing retinal detachment (Table 1, Figs. 3 and 4).

Leave-one-out analysis was performed by analyzing the SNPs associated with IL-15Rα, IL-2Rβ, IL-8 and TSLP by the leave-one-out method, and the results showed that the effect values of the included IVs and the total effect size were relatively close to each other, and the SNPs with a large impact on the causal association estimates had not yet been found (Fig. 5).

*P* values of 0.90, 0.67, 0.17 and 0.33 were obtained by the MR-Egger method and 0.40, 0.52, 0.19 and 0.40 by the IVW method, respectively, and the test of heterogeneity (*P* > 0.05) for IL-15Rα, IL-2Rβ, IL-8 and TSLP indicated that there was no heterogeneity, as shown in Table 2, which was confirmed by the funnel plots, as shown in Fig. 6. In addition, *P*-values of 0.24, 0.35, 0.46 and 0.52 were observed in the MR-Egger regression analysis, which were greater than 0.05, supporting that there was no horizontal pleiotropy among the SNPs and that the instrumental variables did not act through other exposure factors (Table 2).

### Experimental validation of the role of IL-8 in retinal detachment secondary to choroidal melanoma

Lu et al. (2024) identified IL-8 as a risk factor for malignant melanoma (Godtfredsen, 1949). In our study, IL-8 was found to be a risk factor for retinal detachment. Therefore, we need to combine experimental results to verify that IL-8 may be involved in the process of retinal detachment secondary to CM.

**Figure 7:**
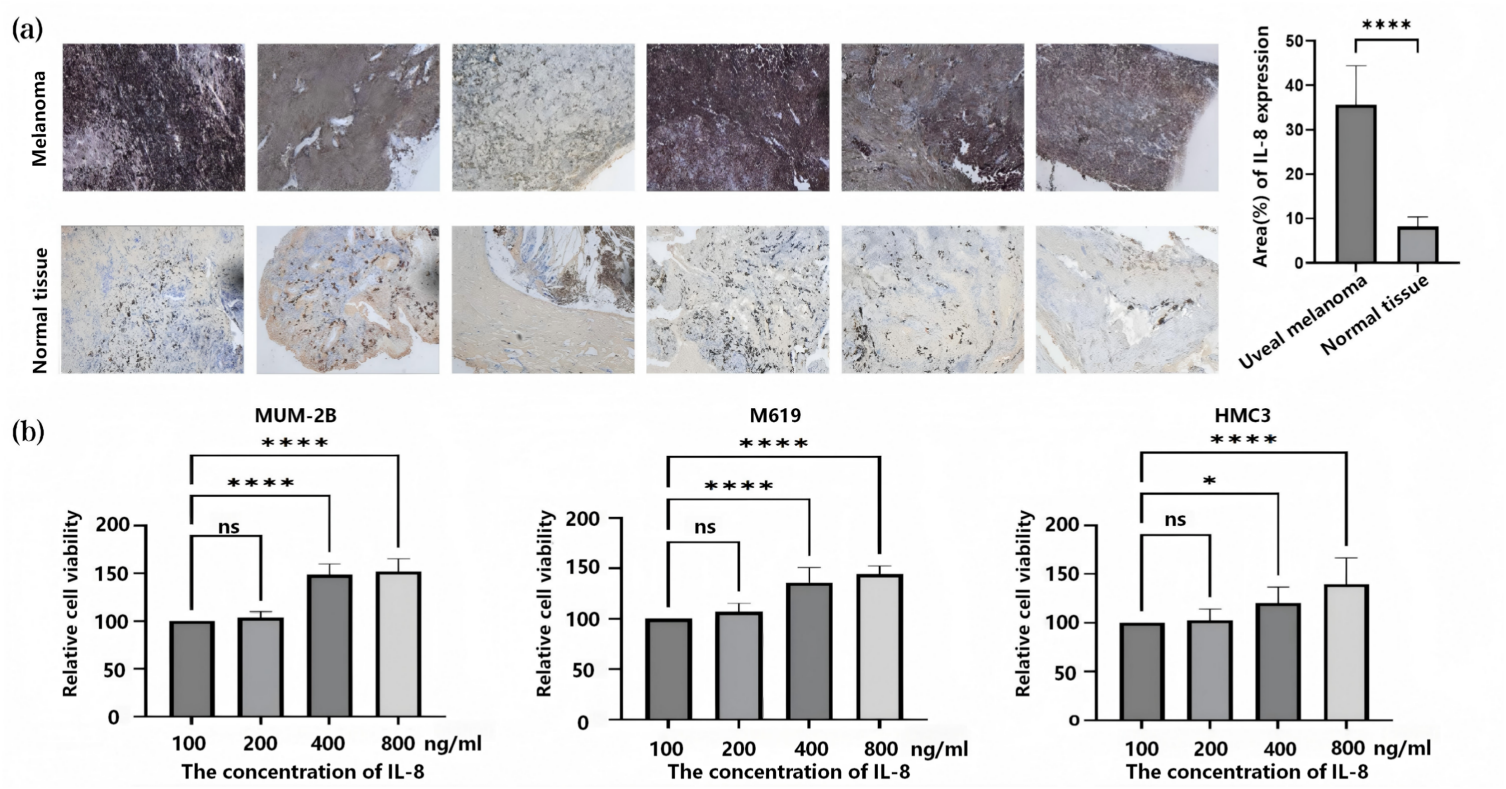
The expression of IL-8 in patients with CM and the effect of IL-8 on cell proliferation. (A) Immunohistochemistry shows IL-8 is highly expressed in human choroidal melanoma tissue as a secretory protein. (B) CCK8. Values represent the mean ± SEM (*n* = 3–6). For comparisons between two groups, a independent samples t-tests is employed. For comparisons involving three or more groups, one-way analysis of variance is utilised. *P < 0.05; ****P < 0.0001. NC, normal control group; ns, no significant difference; SEM, standard error of the mean.

Immunohistochemical analysis revealed that IL-8 is a highly expressed secreted protein in human CM cells and tissues, whereas its expression is low in non-tumour choroidal tissue (Fig. 7A). These findings indicate that CM tumour cells can influence surrounding tissues and cells by secreting IL-8.

**Figure 8:**
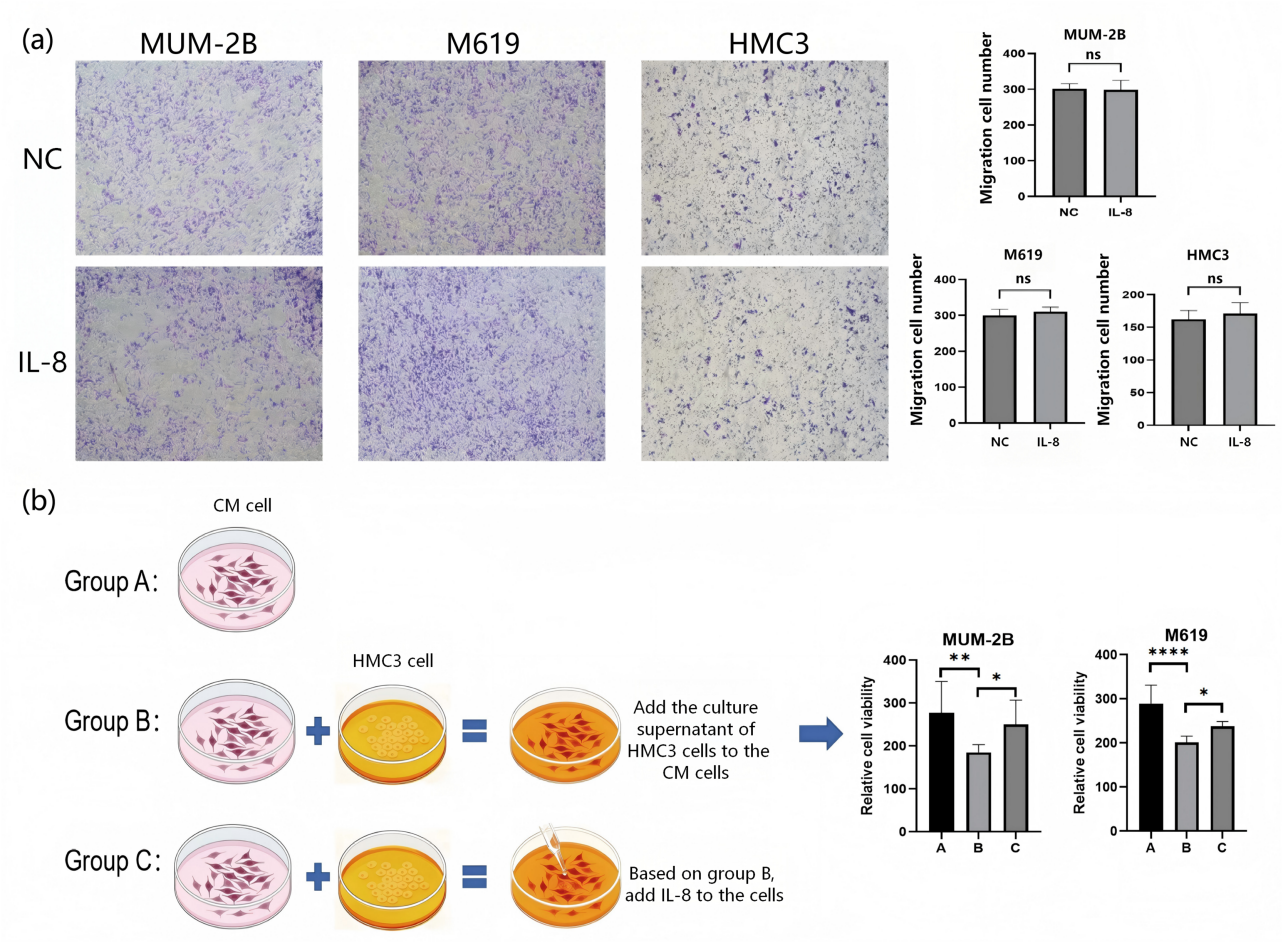
The role of IL-8 in cell migration and its function in the co-culture of HMC3 and CM cells. (A) Transwell migration assay. (B) Co-culture of CM cells with HMC3 cells. Values represent the mean ± SEM (*n* = 3–6). For comparisons between two groups, an independent sample t-test is employed. For comparisons involving three or more groups, one-way analysis of variance is utilised. *P < 0.05; **P < 0.01; ****P < 0.0001. NC, normal control group; ns, no significant difference; SEM, standard error of the mean.

Comparing the growth rates of CM cells and HMC3 cells, no significant difference was observed between the two groups after 24 h of culture without IL-8 or with IL-8 (100 ng/ml) added (*P* > 0.05). However, after 24 h of IL-8 supplementation at concentrations of 400 and 800 ng/ml, the growth rates of both cell lines increased compared to the IL-8-free control group (*P* < 0.05) (Fig. 7B). This demonstrates that IL-8 promotes the growth of CM tumour cells, thereby facilitating tumour progression. HMC3 cells exert immune surveillance within the human eye through mechanisms including cytokine release. These findings reveal that IL-8 promotes their proliferation, potentially exacerbating inflammatory responses and thereby increasing the risk of choroidal melanoma secondary retinal detachment. Furthermore, IL-8 (800 ng/ml) showed no significant effect on the migratory capacity of either CM or HMC3 cells (*P* > 0.05) (Fig. 8A).

In the co-culture experiments, a significant difference was observed between Group A and Group B (*P* < 0.05). However, comparisons between Group A and Group C, as well as between Group B and Group C, revealed that the inhibitory effect of HMC3 cell culture supernatant on the growth of CM cells was weakened (*P* < 0.05) (Fig. 8B). This indicates that the culture supernatant of HMC3 cells has an inhibitory effect on the growth of CM cells, and that HMC3 cells interfere with the growth of CM cells by secreting cytokines. Moreover, the increase in cell viability after the addition of IL-8 suggests that IL-8 may partially alleviate the inhibitory effect mediated by the supernatant of HMC3 cells.

**Figure 9:**
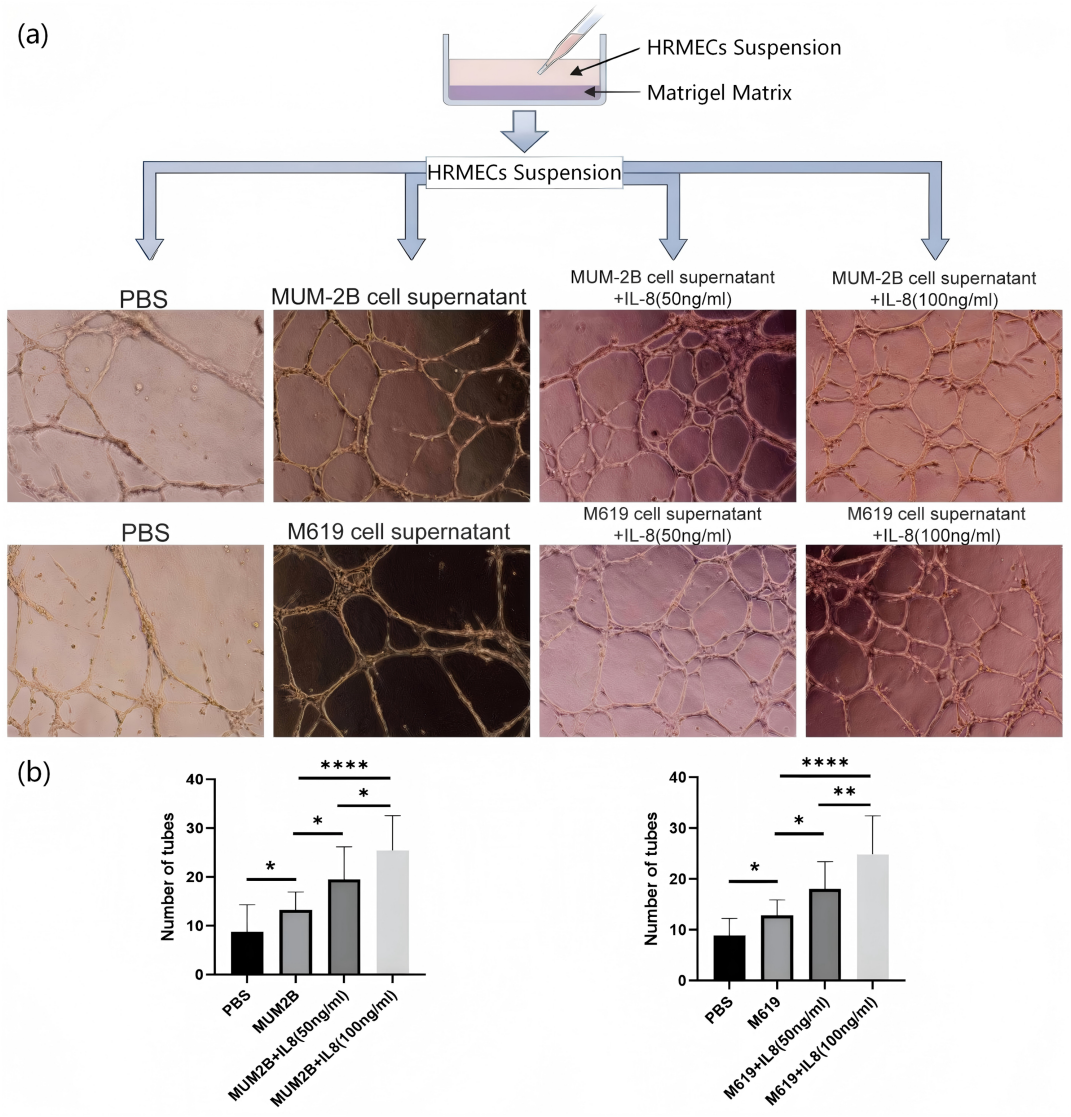
The role of IL-8 in retinal angiogenesis. (A) Tube formation assay. (B) Comparison of the number of tubes formed in each group. Values are expressed as mean ± standard error of the mean (*n* = 3). Comparisons between two groups were performed using independent samples t-test, and comparisons among three or more groups were performed using one-way analysis of variance. *P < 0.05; **P < 0.01; ****P < 0.0001. SEM, standard error of the mean.

The tube formation assay results showed that HRMECs treated with CM cell supernatant and IL-8 both accelerated the formation of blood vessels, and this effect was dose-dependent on the concentration of IL-8 (*P* < 0.05) (Figs. 9A, 9B). Therefore, the CM cell microenvironment plays an important role in promoting the formation of retinal new blood vessels, and IL-8 may exacerbate vascular leakage and promote the progression of retinal detachment. The above results demonstrate that IL-8 is a potential biomarker for retinal detachment secondary to CM.

## Discussion

In the present study, we demonstrated for the first time by MR analysis that CSF-1 and IL-17C were able to increase the risk of malignant melanoma, CDCP1 and IL-10Rβ were able to decrease the risk of malignant melanoma, and that IL-15Rα, IL-2Rβ, IL-8 and TSLP were all able to increase the risk of retinal detachment. Moreover, experimental studies have found that IL-8 is a potential biomarker for CM secondary retinal detachment.

### Causal association of inflammatory factors with malignant melanoma

CSF-1 is a key factor in macrophage differentiation and recruitment of tumor-associated macrophages (TAMs). Its pro-carcinogenic effects are closely related to the immunosuppressive microenvironment mediated by TAMs (Wyrobnik et al., 2023). In this study, we found that elevated CSF-1 concentration increased the risk of malignant melanoma, which may reduce the immune response of malignant melanoma by recruiting TAMs through the CSF-1 signaling pathway, among other methods. In recent years, targeting CSF-1R inhibitors (*e.g*., Pexidartinib) has achieved better results in the clinic (Tap et al., 2019; Palmerini et al., 2020). IL-17C, as a member of the IL-17 family, may promote angiogenesis and tumor cell proliferation through the IL-7/IL-7R-Stat3-IL-17 pathway (Li et al., 2014). Ganzetti G showed that IL-17 expression was higher in malignant melanoma cells than in benign melanocytes (Ganzetti et al., 2015). This is consistent with the results of other studies on the pro-carcinogenic role of IL-17, but the specific mechanism of IL-17C in malignant melanoma needs to be elucidated by functional studies. CDCP1 can promote tumor metastasis by activating the Src kinase pathway (Liu et al., 2011), and high concentrations of CDCP1 may delay the progression of malignant melanoma by inhibiting the expression of matrix metalloproteinases and limiting the immune escape ability of the tumor microenvironment (Miyazawa et al., 2013), which is the same role of the results of this study. IL-10Rβ, as a receptor subunit of the anti-inflammatory factor IL-10, was found to be an important immunomodulatory factor in skin and uveal melanoma cells by Venza I. The IL-10/IL-10 receptor system may be a new target for melanoma therapy (Venza et al., 2015). In this study, we found that its elevated concentration was associated with a reduced risk of malignant melanoma. However, IL-10Rβ often has dual effects in tumor immunity, which still needs to be explored in depth.

### Causal association between inflammatory factors and retinal detachment

IL-15Rα and IL-2Rβ are the receptor subunits of IL-2 and IL-15, respectively, and IL-2 and IL-15 share two receptor subunits and many functions. IL-2 and IL-15 can directly damage adjacent tissues and exacerbate local inflammatory responses by enhancing the survival and activation of T cells and NK cells, and by releasing perforins and granzymes, *etc*. L-8, as a chemokine, is closely related to oxidative stress injury and extracellular matrix remodeling. Oxidative stress injury and extracellular matrix remodeling are closely related (Choi et al., 2019; Waldmann, 2002). Some studies have found that the most important role of IL-8 in the eye is angiogenesis and induction of ocular inflammation (Ghasemi et al., 2011). TSLP is a pleiotropic cytokine that plays a key role in the onset and development of allergy and asthma. It is mainly produced by epithelial cells and is a potent immune system activator. It has been found that TSLP activates the JAK-STAT signaling pathway in B cells, which promotes inflammatory cell aggregation and further increases vascular permeability (Lu et al., 2022). TSLP also promotes a Th2-type immune response, recruiting eosinophils to release eosinophil cationic proteins (ECPs), which exacerbates inflammatory responses (Hammad & Lambrecht, 2015; Ito, Liu & Arima, 2012). Therefore, the present study found that elevated concentrations of IL-15Rα, IL-2Rβ, IL-8 and TSLP increased the risk of developing retinal detachment, possibly due to increased vascular permeability caused by local inflammation or neovascularization, which led to the accumulation of subretinal fluid. However, there are no studies on the mechanisms associated with retinal detachment and IL-15Rα, IL-2Rβ, IL-8 and TSLP, and further exploration is needed.

### Causal association of inflammatory factors with choroidal melanoma secondary to retinal detachment

IL-8 is implicated in the progression of both CM and retinal detachment. Previous studies have demonstrated that IL-8 modulates melanoma cell proliferation, migration, and induces angiogenesis by stimulating extracellular matrix-degrading enzymes, which are pivotal for melanoma growth and metastasis (Singh & Varney, 2000). The inflammatory response and neovascularisation induced by malignant melanoma can lead to increased tissue fluid leakage and accumulation of exudate (Shi et al., 2023b, 2023a). Furthermore, several clinical studies have confirmed a positive correlation between IL-8 levels in the vitreous humour of retinal detachment patients and the severity of retinal detachment (Rasier et al., 2010; Takahashi et al., 2016). Lu et al. (2024) identified IL-8 as a risk factor for malignant melanoma in a two-sample Mendelian randomisation study involving 41 inflammatory cytokines. In the present study, IL-8 was found to be a risk factor for retinal detachment. Although this Mendelian randomisation study exclusively enrolled European populations, numerous investigations have demonstrated that IL-8 constitutes a significant ocular inflammatory factor in Asian cohorts and correlates with retinal detachment (Ghasemi et al., 2011; Takahashi et al., 2016). Consequently, IL-8 may represent a pivotal inflammatory mediator in choroidal melanoma-associated retinal detachment.

Meanwhile, immunohistochemical analysis revealed that IL-8 is highly expressed as a secreted protein in CM tissue. Cellular functional experiments indicated that IL-8 enhances the proliferation of CM cells and HMC3 microglial cells, partially reduces the growth inhibition effect of microglial cell culture supernatant on CM cells, and promotes endothelial tube formation in a dose-dependent manner. Some studies have found that activated HMC3 cells can release IL-8, and IL-8 can promote the migration of HMC3 cells. However, there are few findings about how IL-8 promotes the growth of HMC3 cells. Lattanzio et al. (2013) found that, apart from VEGF, IL-8 is a pathway that maintains the angiogenesis of uveal melanoma. This finding is consistent with the results of the current study (Shu et al., 2023; Du et al., 2025; Dandan-Zong et al., 2023). These findings may construct a model where IL-8 released by CM leads to the strengthening of a pro-tumor inflammatory microenvironment. This could weaken the dysregulated interaction between microglia and tumors due to impaired local immune surveillance and enhance pro-angiogenic signaling that increases vascular permeability. Notably, this provides a direct pathway to the hallmark characteristic of exudative retinal detachment in CM—accumulation of subretinal fluid. Importantly, our study not only reaffirms the elevated IL-8 levels but also confirms its role through genetic causality. It directly links IL-8 to immune regulation by microglia and vascular leakage-related angiogenesis, thereby revealing the mechanism underlying the extensive exudation and poor visual prognosis observed in some CM patients.

From a clinical perspective, IL-8 may provide a theoretical basis for targeted anti-inflammatory or combined anti-angiogenic therapy. Several IL-8 inhibitors currently demonstrate effective blockade of IL-8-related pathways and inflammatory responses (Bilusic et al., 2019). Bilusic et al.’s (2019) research identified HuMax-IL8 (BMS-986253) as a novel fully human monoclonal antibody capable of inhibiting IL-8, exhibiting safety and tolerability. Clinical evaluations are underway to assess the efficacy of combining IL-8 blockade with other immunotherapies. Villa et al.’s (2007) research demonstrated that the IL-8 (IL-8/CXCL8) receptor inhibitor Reparixin ameliorates neurological deficits and reduces chronic inflammation in rats subjected to permanent and transient cerebral ischaemia. He et al.’s (2021) research indicates that the IL-8 antagonist SB225002 significantly delays tumour growth, eliminates tumour-associated neutrophil (TAN) accumulation, and mitigates inflammatory responses. IL-8 may serve as a key biomarker for retinal detachment secondary to choroidal melanoma, with these inhibitors offering valuable reference points for future clinical treatments and research.

IL-8 can be integrated into clinical practice as a measurable biomarker to support the monitoring of CM-related exudative complications. In clinical practice, IL-8 levels in the ocular fluids of CM patients, such as aqueous humor and vitreous, can be quantified using routine immunoassays like ELISA. Additionally, serum IL-8 levels in CM patients could serve as a less invasive alternative marker and require prospective validation. By identifying CM patients at higher risk for subretinal exudation and retinal detachment through these methods, combining traditional CM therapies with IL-8 inhibition strategies could help control disease progression and improve patient outcomes.

### Strengths and limitations of the study

The strengths are (1) Using a two-sample Mendelian randomization design, we systematically evaluated the causal associations of 91 inflammatory factors with malignant melanoma and retinal detachment, and found that IL-8 is a risk factor for retinal detachment. This study broke through the confounding biases of traditional observational studies. (2) The robustness of the results was ensured by multi-method validation (IVW, MR-Egger, *etc*.) and rigorous screening of instrumental variables, revealing for the first time the genetic effects of multiple influential factors. (3) The study covers a wide number of inflammatory factors and more significant targets are already available as clinical drugs, providing a precise intervention basis with high translational potential for anti-inflammatory therapy.

Limitations are (1) GWAS data were mainly derived from European populations, and there may be racial differences in causal associations; (2) MR reflects lifetime genetic effects and cannot capture the dynamic changes of inflammatory factors during the progression or treatment of malignant melanoma and retinal detachment; (3) This study did not directly incorporate CM subtype data, potentially introducing bias in causal association results; (4) Research on IL-8’s mechanism of action remains limited and warrants further investigation.

## Conclusion

This study identified IL-8 as a risk factor for retinal detachment from a genetic perspective. Moreover, IL-8 was found to be highly expressed in CM tissue, where it can promote the progression of CM, inflammatory responses and angiogenesis. Therefore, IL-8 is a potential biomarker for retinal detachment secondary to CM and provides a new therapeutic target for targeted anti-inflammatory treatment.

## Supplemental Information

10.7717/peerj.21183/supp-1 Supplemental Information 1 Raw Data.

10.7717/peerj.21183/supp-2 Supplemental Information 2 Code.

## Additional Information and Declarations

### Data Availability

The following information was supplied regarding data availability:

The raw data and code are available in the Supplemental Files.
